# The Philanthropist's Vade Mecum

**Published:** 1890-01-04

**Authors:** 


### The Philanthropist's Vade Mecum.

### GENERAL HOSPITALS.

**Charing Cross Hospital,** standing at the entrance to the Strand, and near the great station of the same name, is peculiarly liable to be called on to open its doors for the admission of sufferers from the accidents so common in our London streets. Although partly endowed, it is in urgent need of money to extend the medical school attached to it. Lest the charitable should think that this is no concern of theirs, it may be mentioned that the students' fees form no inconsiderable part of the revenue of the hospital. It is also desired to establish a convalescent home in connection with the hospital, as only the change to country air will complete the cure begun within its walls.

**French Hospital,** situated in the appropriate neighbourhood of Leicester-square, is open without payment or recommendation of any sort to all the sick who can speak the French tongue. As it was in 1789 that the first great influx of distressed foreigners came across the Channel, we suggest to those who have lately been waving the red cap of liberty in Paris and elsewhere, that a contribution to this hospital would be an eminently suitable way of celebrating the centenary of the French Revolution.

**Great Northern Central Hospital,** Holloway-road, N. Secretary, Mr. W. T. Grant.—This is a free hospital where patients are admitted without letters of recommendation. It was formerly the Great Northern Hospital, in the Caledonian-road, but was removed to the new buildings in Holloway-road in January, 1888. It at present contains sixty beds, but the new hospital, when completed, will contain 150 beds. It is to be hoped that funds will be provided to enable this to be done during 1890, seeing that the necessities of the poor of North London demand the whole of the 150 beds, without which much needless and avoidable suffering must result to the poorer population. If some millionaire would take this hospital under his patronage and complete the new buildings at his own cost, he would render better service to his day and generation than a similar amount of money could purchase in other directions. We would ask those of our readers who have large means at their disposal to visit this institution, and then to drive round the district in which it is situated. If they do this we have little doubt that the whole of the hospital buildings will be erected and opened without delay. Prince Albert Victor has recently become president, a fact which emphasises the importance which the Royal Family attach to the work of this institution.

**Italian Hospital,** in Queen's-square, is an institution of modest pretensions, but as so many English go to Italy in search of health, it would be but a fair expression of gratitude on their part to return the compliment by a donation to the funds of an institution whose primary object is the relief of Italians who have the misfortune to fall ill in London.

**King's College Hospital,** lying in the retirement of Lincoln's-inn-fields, is better known to the suffering poor than to the charitable public. With some of the most famous physicians and surgeons of the day on its staff, with nursing arrangements in the highest degree admirable, it has not met with such an amount of public favour as to enable it to keep its head above water. From the back streets which lie between Shaftesbury-avenue and the Strand it drew last year nearly 2,000 in-patients, and over 8,000 out-patients, besides 8,000 casualties. There is more work for it to do, and it would gladly do more if the governors had not been compelled to close two wards for lack of funds to maintain them. Any subscriptions given to this hospital will be a genuine boon, not merely to those now treated within its walls, but to medical science in general.

**London Hospital,** with its 800 beds, large as it is, does not by any means offer more accommodation than is needed by the sick and suffering of the East-end. The out-patients last year numbered nigh upon 100,000, and the in-patients were nearly 8,000. These were maintained at an expenditure of over £30,000, of which little more than a fourth is assured by endowment. At a time when the East-end is prominently before the public eye, it is hardly necessary to appeal for help on behalf of this, one of the noblest of our charitable institutions.

**London Temperance Hospital,** Hampstead-road, N.W. Secretary, Mr. Tom Mundy. This is a well-managed hospital, conducted on temperance principles, and is well worthy of a visit. The patients are most comfortable, the treatment is in every way satisfactory, and the results are encouraging. Anyone can endow a bed by contributing a lump sum of £1,000, and we wonder how it is that so few of the many thousands of devoted people who believe in temperance should have neglected this golden opportunity of emphasising and enforcing the principles which they advocate. There are altogether seventy beds, and upwards of 3,000 patients are treated annually.

**Metropolitan Hospital,** Kingsland-road, E. Secretary, Mr. C. H. Byers.—This hospital is worthy of all support, being conducted on provident principles, and so setting an example to its fellows throughout the metropolis. It has no endowment, and, although there are 160 beds, many of the wards are vacant for want of funds. The hospital is situated in a poor district, and it is to be hoped that Sir Edmund Currie may be enabled during the year 1890 to so finance it as to enable the whole of the beds to be made available for patients.

**Middlesex Hospital** contains 300 beds, of which thirty-four are set apart for the reception of cancer patients. The long care and general treatment required for sufferers from this terrible disease add greatly to the expenses of the hospital. So little accommodation is provided anywhere in the kingdom for sufferers from this most incurable of ailments, that this should be an inducement to the charitable in all parts of the country to contribute something towards the funds of this beneficent institution.

**Poplar Hospital for Accidents,** Blackwall, E. Secretary, Lieut.-Colonel Feneran, 303, East India Dock-road, E.—This is practically the only exclusively accident hospital in the whole of London. Need anything more be said to secure for it adequate support? It contains fifty-one beds, and treats nearly 10,000 patients yearly.

**Royal Free Hospital,** Gray's-inn-road, is one of the few institutions where a governor's letter is not required to obtain admission. Poverty and suffering are the only recommendations needed. Being situated near the great railway termini, a large number of accidents are brought to its doors. Besides these, it stands in a neighbourhood that is both crowded and busy. The number of in-patients treated annually amounts to over 2,000, and the out-patients to 25,000; while, to meet their wants, the reliable income of the hospital is little more than £1,500. How much this leaves to be filled by voluntary charity is apparent to the dullest mind. The hospital can boast economy among its other virtues, when it shows that the annual expenditure is only about £11,000; but the necessity of living from hand to mouth throws a great and needless amount of anxiety upon those responsible for its management.

**Seamen's Hospital (Dreadnought),** Greenwich. Secretary, Mr. P. Michelli.—When we state that this hospital is free to seamen of all nations, regardless of race, creed, or colour, will not most people be inclined to send a contribution to so cosmopolitan a charity? There are two free dispensaries for seamen, one at the London Docks, and one at Gravesend, whilst a branch hospital is being built in the centre of the Docks, so as to lessen as much as possible the sufferings of those who meet with accidents in the Port of London.

**St. George's Hospital**, standing at Hyde Park Corner, has been credited with being a wealthy institution. It would be rash, however, to assume that all the wealthy people who pass its doors contribute to its funds, and we gladly take this opportunity of recommending an institution which has often been helpful to those injured in our crowded thoroughfares to the goodwill and purses of rich and poor.

**St. John and St. Elizabeth's Hospital**, Great Ormond-street, affords a home for those chronic cases which are unsuitable for general hospitals. Many authorities are of opinion that the supply of incurable hospitals is still insufficient, and that both hospitals and the poor suffer for lack of them. If this be so, it is all the more evident that it is the duty of all to give some measure of support to such as we have.

**St. Mary's Hospital** is the only general hospital in the district of Paddington—a district which contains nearly a quarter of a million inhabitants. To meet this the total number of beds available in the hospital is 280, and the governors are very anxious to extend the size and increase the usefulness of the institution. A suitable site has been obtained, but unless ample subscriptions are obtained the enlarged hospital will be sorely burdened with debt.

**University College Hospital** is a totally unendowed institution. It is practically free, as no suitable applicant is rejected while there is a vacant bed. It treats on an average about 3,000 in-patients and 15,000 out-patients annually, while attending many cases in the homes of the poor. With a constantly increasing demand on its accommodation, it is too small for the thickly-populated district in which it stands, and rebuilding upon a more modern plan is urgently necessary. The funds already promised for this purpose amount to over £11,000, but the committee, who believe that debt is a danger, are afraid to begin operations until that sum is nearly trebled. We commend this hospital to those who, living in the neighbourhood, may profit by its accommodation, or may some day wish to send their sons to the medical school attached to it.

**Westminster Hospital**, standing in the shadow of the ancient abbey, possesses a certain assured income, which, however, amounts only to a fifth of the annual expenditure, and recently the authorities have been obliged to spend a considerable sum in new buildings. As the number of casualties received within its walls numbered nearly 8,000, it may be inferred that the hospital has a claim on the sympathies and purses of others besides the residents in its neighbourhood. The crowds who go to listen to popular preachers at the Abbey or St. Margaret's, besides the visitors to the large hotels near, would do well to consider the wants of this hospital, which is a Broad Sanctuary to the poor and distressed.

### HOSPITALS FOR CONSUMPTION.

**Hospital for Consumption and Diseases of the Chest**, Brompton. Secretary, Mr. Henry Dobbin.—As we have said on many previous occasions, London has far too few hospital beds for the treatment of cases of consumption. This hospital was established so long ago as 1841, and when we state that its expenditure exceeds its income by nearly £2,000 a year many people will, no doubt, be glad of an opportunity of contributing liberally to the impoverished exchequer of so old and so deserving an institution.

**North London Hospital for Consumption**, Mount Vernon, Hampstead. Secretary, Mr. Lionel F. Hill, 216, Tottenham-court-road, N.—This hospital was entirely reconstituted a few years ago, when the residents at Hampstead manifested considerable interest in the welfare of the institution, which has been attended by the happiest results. Thanks to Mr. Benjamin Lyon, the chairman, and the other members of the committee, a very genuine interest is displayed in the work. Further and much-needed extension are in contemplation, and the annual dinner attracts more people and is certainly more enjoyable than probably any other charity dinner of the year. All this points to efficiency, and should secure liberal contributions to enable the managers to largely extend the good work they have in hand.

**Royal Chest Hospital**, City-road, E.C. Secretary, Mr. Harrold.—Recent events have called public attention to this charity to an unusual extent, and we hope that the increase in the annual subscriptions and other contributions which have taken place may be maintained and extended in the year 1890. Mr. Harrold, the new secretary, has a stiff piece of work before him, but his record justifies the belief that he will accomplish it successfully. This hospital provides treatment not only for sufferers from our national scourge, consumption, but for those acute forms of chest disease in which prompt care means recovery and neglect a lingering death. Situated in such a district as the City-road, the demands made on its accommodation both by in and out-patients, but especially the latter, are large and pressing, and with an increased income it would gladly develop into increased usefulness.

### HOSPITALS FOR CHILDREN.

**Alexandra Hospital for Children with Hip Disease** forms a refuge for many little sufferers who are called on to endure long periods of pain, often with but little hope of cure. The demand made on the resources of the hospital is evidenced by the fact that generally the whole number of available beds are occupied, although the little patients are sent in turn to the branch hospital at Bournemouth. Those who love to see healthy children play might well contribute something to an institution which takes care of those to whom active play is impossible.

**Cheyne Hospital**, Cheyne-walk, Chelsea, S.W. Matron, Miss Elam.—This hospital is invaluable, from the circumstance that it provides for incurable children as well as for those who cannot be retained long enough in general hospitals to give them a chance of permanent relief or of ultimate recovery. There are twenty-four beds, the whole of which were occupied throughout the year, a circumstance which emphasises the importance of the work, and proves the necessity for the liberal gifts of the charitable public to enable the managers to largely extend it.

**East London Hospital for Children and Dispensary for Women**, Shadwell, E. Secretary, Mr. Ashton Warner; lady superintendent, Miss F. A. Davies.—This hospital was originally established in Ratcliff-highway, and we shall never forget our visit to the old warehouse in which it was originally located. During the time it was occupied as a hospital Charles Dickens made it famous by the graphic description he wrote of the institution in those days, and our experience and knowledge enable us to urge philanthropists to examine its work with the view to becoming permanent subscribers of one of the best of our children's hospitals. Mr. Ashton Warner is indefatigable, and has the advantage of the support of a devoted staff, whose zeal and efficiency are universally recognised. The institution contains upwards of one hundred beds, and treats about 15,000 out-patients annually. A donation of £1,000

will provide for the permanent endowment of one cot, whilst the sum of £300 will enable the giver to endow a cot for the whole period of his own life. Each cot is estimated to cost £50 per annum.

**Evelina Hospital for Children** has in its whooping cough ward a beneficial speciality, which is not to be met with in any other of the children's hospitals in London. It also receives infants of more tender years than are generally admitted elsewhere. It stands in a district whose resident inhabitants may nearly all be numbered among the poor, although the manufactories and warehouses of the neighbourhood represent no little wealth. To the sympathies of the latter we commend this useful institution.

**North-Eastern Hospital for Children,** 326, Hackney-road, E. Secretary, Mr. Alfred Nixon.—The financial condition of this hospital shows what can be done in London to-day for a medical charity by an energetic secretary who understands his business and determines to carry it on with zeal and devotion. In addition to the treatment of children at the hospital, it maintains convalescent homes at Croydon (Surrey) and Brandon (Norfolk), which are under the direction of a ladies' committee. There can be no doubt that of all institutions the Children's Hospital affords the very best field for the work of a ladies' committee, providing the members of it are capable and show much discretion in their proceedings. The fact that nearly £800 a year is received from patients' payments towards an expenditure of about £5,000 should encourage intelligent givers to subscribe liberally to the funds.

**Paddington Green Children's Hospital** does a modest but useful work in a district where children are numerous and accidents not few. As the hospital in Great Ormond-street is the nearest institution of a similar character to that on Paddington Green it will be seen that the latter fully justifies its existence, and only seeks for means to justify it to a still larger extent.

**Victoria Hospital for Sick Children,** Queen's-road, Chelsea, S.W. Secretary, Commander Blount, R.N.—In addition to the hospital proper, there is a convalescent branch at Margate with fourteen beds. The hospital contains seventy beds, which are usually full, and relieves upwards of 10,000 out-patients every year. Owing to the zealous care of those connected with this charity, overwhelming debt is avoided, a fact worthy of encouragement as pointing to efficiency and scrupulous care in the management. There are thirteen endowed cots, but the committee would be very glad if the number could be at once increased to, at least, twenty. This institution should commend itself especially to the support of the many wealthy and well-to-do people who reside in the district where it is situated.

### HOSPITALS FOR WOMEN.

**Chelsea Hospital for Women** had for many years a hard struggle for existence, and though it has now entered on a more prosperous career, its needs are still in excess of its income. It receives paying patients, as well as those recommended by subscribers; but as some of these contribute less than half the cost of their maintenance, voluntary subscriptions are still required. We commend this institution to all who realise how much the health of woman affects the happiness of home.

**New Hospital for Women,** Marylebone-road, is a living proof that many women prefer being attended by physicians of their own sex. There are now far more patients asking for admission than can be received, and it is therefore necessary to build a new hospital now that funds permit. As, however, only one bed in the hospital is endowed, much money must be obtained by subscriptions and donations before the contemplated enlargement can be proceeded with as cheerfully as would be desired.

**Samaritan Hospital for Women and Children,** Lower Seymour-street, Portman-square, W. Secretary, Mr. George Scudamore.—This hospital has been the scene of the successful labours of Sir Spencer Wells, who has done more than anyone else to prolong the life of English women, and, indirectly, of women all the world over. Some 800 ovarian operations have been performed at this institution, with a mortality of less than 1 in 10, a truly remarkable record, which should secure an abundance of funds for the continuance of a work of national importance.

**Grosvenor Hospital for Women and Children,** Vincent-square, S.W. Superintendent, Miss K. Hughes; hon. secretary, Mr. A. J. Ram.—We have heard a good deal to the credit of this institution during the last year, and are glad to know that its work is progressing satisfactorily and well. It contains fourteen beds, and relieves between two and three thousand out-patients. It is founded on the principle that patients should contribute to the maintenance of the hospital according to their means. These contributions at the present time are sufficient to defray upwards of one-third of the expenditure.

### LYING-IN HOSPITALS.

**Queen Charlotte's Hospital,** Marylebone-road, does a good and necessary work in giving a home to poor women at the time when they most need kind care and skilful treatment. Besides this, it gives admirable training to nurses and midwives, who go forth to aid the rich as well as the poor in their time of trouble. To all wives and mothers we commend this institution, which does so much for their less fortunately-placed sisters.

**City of London Lying-In Hospital,** City-road, E.C. Secretary, Mr. R. A. Owthwaite.—Originally established in 1750, it now contains thirty-four beds, and relieves upwards of 1,600 patients per annum. It acts also as a school for the training of midwives, and as its management is above suspicion, we hope Mr. Owthwaite will receive a fair proportion of the New Year's offerings of the charitable. We especially commend the Samaritan Fund of this charity, which does great good amongst the poorer patients on their leaving the hospital.

### NERVOUS DISEASES, EPILEPSY, AND PARALYSIS.

**Hospital for Epilepsy and Paralysis,** 32, Portland-terrace, Regent's Park. Secretary, Mr. H. Howgrave Graham.—This institution admits cases of paralysis and epilepsy on payment or without. The ordinary expenditure, exclusive of investments, is about £2,000 a year, of which about one-third is derived from patients' payments. The George Sturge Samaritan Fund, established in 1884, is devoted to the relief of destitute patients. There are twenty-five beds, and about ninety patients are relieved annually. The secretary has shown much energy and ability in developing the resources of this institution; its report is one of the most interesting issued from the press, and we should be glad to hear that more funds have been placed at the disposal of the committee.

**National Hospital for Paralysed and Epileptic,** Queen-square, W.C., is an altogether unique institution. Its patients are drawn from every quarter of the kingdom, and are suffering from some of the most fearful diseases that afflict humanity. The highest triumphs of modern surgery and the utmost skill of modern nursing are required to effect a cure of the cases there treated. To cure, or even permanently relieve, such cases, a large expenditure both of skill and money is needed, for though the weekly cost of each in-patient is by no means excessive, a long residence in the wards is necessary for a beneficial result to be obtained. A satisfactory feature of the hospital is that about a fifth of its income is derived from patients' payments, but as the poor suffer from paralysis as well as the rich, there is ample room for the operation of charity.

### CANCER.

**Cancer Hospital**, Brompton, is devoted to the treatment not only of those hopeless cases from which it takes its name, but of many species of tumours which if neglected might prove fatal, but if dealt with in time restore many useful lives to the community. It is entirely free, and last year treated 700 in-patients, while over a thousand out-patients attended for advice and medicine. The annual expenditure of £25,000 may seem large in proportion to the cases treated, but as the patients spend many months, sometimes years, in the hospital before death or cure relieves them, it cannot be considered excessive, for in such cases economy must needs go hand in hand with comfort.

### HOSPITALS FOR INCURABLES.

**British Home for Incurables**, 380, Clapham-road, S.W. Secretary, Mr. R. G. Salmond, 73, Cheapside, E.C.—Funds are greatly needed at the present time by this institution, which has found it necessary, owing to the expiration of the lease of the present building, to proceed with the erection of a new hospital. The difficulties of maintaining an institution of this kind can be realised from the fact that the secretary, Mr. Salmond, was entirely broken down by the work at the beginning of the year. We are glad to be able to congratulate him upon his recovery, and take this opportunity of expressing a hope that he will long be spared to extend the usefulness and maintain the efficiency of this great institution. The present buildings contain seventy beds, and there are in addition upwards of 200 out-pensioners. These pensioners receive an annuity of £20 a year, and are thus enabled to continue to reside with their friends or relatives, thereby keeping the family together, and promoting the mutual happiness of all concerned. We cordially commend the work of the British Home for Incurables to all who are able to indulge in the luxury of giving liberally at this season to charities of proved usefulness and value.

### MISCELLANEOUS SPECIAL HOSPITALS.

**Lock.**

London Lock Hospital and Asylum, Harrow-road, has not met with the support it deserves. A foolish and most unchristian prejudice exists against institutions of the sort —a prejudice which would be at once dispelled were it more generally known that of the 600 patients who annually enter this hospital more than a fourth pass into the rescue home and are reclaimed from their lives of misery.

**Ophthalmic.**

Royal London Ophthalmic Hospital, Moorfields, does an excellent work in the prevention as well as the cure of diseases of the eye. In these days of educational over-pressure many children are afflicted with ailments which, generally classed under the title of short sight, are due to many different causes. To such as these the existence of such a hospital as that at Moorfields is an undoubted boon. The out-patients naturally out-number largely those within its walls—last year they were 25,000—but a hundred beds are available for those who need prolonged care and constant attention. Of all the ophthalmic hospitals in London, there is none that so well deserves the generous consideration of the public.

### DISPENSARIES.

**Finsbury Dispensary**, Goswell-road.—This is one of the oldest dispensaries in London. It has the great advantage of possessing as hon. secretary Mr. R. Moreland, who is working energetically on its behalf. There is to be a banquet on the 11th of March next, in the Grand Hall of the Freemasons' Tavern, at which the Lord Mayor, the Right Hon. Sir Henry Isaacs, will preside, supported by the sheriffs and many influential personages. A hundred years ago the festival dinner of this charity was able to attract upwards of 300 guests. We wonder if it will be possible to assemble anything like this number at this year's dinner. If so, it will be one more instance of the power which a good example exercises over others. In any case, the Finsbury Dispensary is an excellent charity worthy of liberal and sustained support. We wish it all success and prosperity, and take this opportunity of thanking Mr. Moreland for his self-denying efforts in so good a cause.

### NURSING INSTITUTIONS.

We specially desire to commend to the generous consideration of the charitable the many nursing associations in various parts of London which provide nurses for the sick poor. There is no branch of work more worthy or deserving than this, and it is desirable that it should be encouraged by increased liberality on the part of the public. We hope that our readers may be induced to send contributions to one of the following:

Bible Woman Nurse, 2, Adelphi-terrace, Strand, W.C. Hon. sec., Mr. G. Selfe Leonard.

East London Nursing Society, 49, Philpot-street, Commercial-road, E. Secretary, A. W. Lacy.

Metropolitan and National Nursing Association, 23, Bloomsbury-square, W.C. Hon. inspector of nursing, Mrs. Dacre Craven; hon. sec., Rev. Dacre Craven.

North London Nursing Association, 413, Holloway-road, N. Superintendent, Miss De Lüttischan.

Nursing Sisters of St. John the Divine, 68 and 70, Drayton-gardens, South Kensington, S.W. Sister Superior, Selina S. Wilson.

Workhouse Infirmary Nursing Association, of 6, Adam-street, Strand, is one of the best of our charitable nursing institutions. It has worked a perfect revolution in the workhouse infirmaries throughout the country, and the dying pauper is now sure of a certain amount of care and attention when his last trial has arrived, and he has to face the valley of the shadow of death. The association has fought its way nobly against strong prejudices; it has been a pioneer society, and therefore has had a hard path to travel, and it still needs support and increased funds. There yet remain infirmaries where the sick and dying are trusted to the tender mercies of imbecile paupers, and if only the association had larger funds this would soon be remedied. The association has an income of about £300 a year, and the amount of work it does with this money is simply extraordinary.

### A FEW OTHER CHARITIES.

**"After-Care" Association,** for poor and friendless female convalescents who leave asylums for the insane, does a unique and most valuable work in giving help and shelter to those who, though no longer insane, are not fit, without some little preparation, to take their place on the battlefield of life. During the last year fifty cases have been treated by the association, some being sent to convalescent homes, and others helped by gifts of money and clothing, or the still more precious boon of suitable employment. The fact that the Earl of Meath is president of the association, and that it has the support of the medical superintendents of many important asylums is a sufficient guarantee of its usefulness. The secretary is Mr. H. Thornhill Roxby, Aiden Lea, Walthamstow.

**Christian Community,** Memorial Hall, London-street, Bethnal Green, E. Secretary, Mr. James Atkinson.—This organisation was originally established by the Huguenots in 1685, and was subsequently reorganised by John Wesley in 1772. Its operations for the past year included the visiting of workhouses, casual wards, and lodging-houses. In the summer months children and adults are taken into the country, and are maintained there for a week. It has 400 voluntary workers associated with it, of both sexes, who give their time gratis to the work of the society. It provides thousands of homeless people with all-night shelter and breakfast; dinners are provided for children, and bread, lodging, and coal tickets are distributed throughout the winter. There are also mission-halls and numerous services of various kinds in addition to a home for working girls, where the inmates are trained for service, being afterwards placed in suitable situations. On the whole, as the late George Moore said, "The real work of this mission beats all I have yet met with in my experience, as I know nothing like it in simplicity and downright earnestness in going amongst the very lowest and poorest classes, so that everybody must desire to help the Christian Community in every possible way."

**City of London Truss Society,** 35, Finsbury-square, E.C. Secretary, Mr. John Whittington.—The work of this society is unobtrusive, but eminently practical, supplying, as it does, instruments for the relief of the ruptured poor throughout the kingdom. Perhaps, as a general rule, the sympathies of the philanthropic public are more easily attracted by the claims of hospitals and institutions devoted to relieving the sick. But it should not be forgotten that a very substantial proportion of human beings, though healthy in other respects, are in constant need of such instruments as this society supplies. Without them their lives would be a burden, and their earning powers seriously impaired. The Bishop of Derry scarcely exaggerated when he said that the City of London Truss Society was "one of the most useful and excellent institutions in existence," and we hope it will receive a fair share of patronage this season.

**Factory Lads' Home,** 331, Kentish Town-road, N.W., aims at bringing wholesome influences to bear upon boys at the time when they leave school and go out into the world for themselves, without any adequate knowledge of its dangers, while they are peculiarly susceptible to its temptations. To give them innocent amusement and helpful instruction which will keep them off the streets, is the object of this institution.

**London Female Penitentiary and Guardian Society,** 191, High-street, Stoke Newington, N. Secretary, Mr. W. Edwin Page.—This, one of the oldest penitentiaries in the country, is under the patronage of Her Majesty the Queen. It was founded at Pentonville, 1807, and now consists of a training home at Stoke Newington, with seventy inmates, and a probational home in Old Ford-road, E., with accommodation for a similar number. There is no endowment nor invested funds, and the institutions are entirely dependent upon voluntary support. The society is unsectarian in constitution and management, and its work is of national importance. Of the women received into the home during the first year, two-thirds did not exceed twenty-one years of age, and thirty-two of the applicants were not more than eighteen years. The society has tried by simple religious instruction and judicious moral and industrial training to fit the objects of its care for situations of usefulness and respectability. It feeds, clothes, and trains in industrial work all who are thus afforded an opportunity for a fresh start in life. Lord Shaftesbury was one of its warmest friends and supporters, and nearly ten thousand young women have already been benefited through this institution. It may be added that the words "Guardian Society" better describe its work than Female Penetentiary, seeing that it is in no sense a place of detention, the mere mention of such a system being repellent to the managers and the lady superintendent (Mrs. E. Walter).

**Mrs. Hilton's Creche** at Stepney-causeway takes in daily between 80 and 100 children, whose ages vary from three weeks to five years. The shelter thus provided for their babies sets many mothers free to go out and earn their living without any anxiety as to what is going on at home. An infant home is attached to the crèche for the purpose of taking charge of children whose mothers are in hospitals. There can be little doubt that such institutions do much to moderate the high rate of infant mortality, and all who are touched by the helplessness of childhood would do well to send a contribution to Mrs. Hilton.

**National Orphan Home,** 12, Pall-mall, S.W. Secretary, Mr. E. Evans Cronk.—The appeal which the committee issues is for £500, to enable them to meet current liabilities and make necessary repairs at the Home. The building is at Ham Common, near Richmond (Surrey), and its object is to shelter fatherless girls, who are educated and trained for domestic service. So practical an institution scarcely needs special recommendation from us, for the difficulties and temptations that beset young and friendless girls alone set up a claim for assistance, that falls almost as a duty upon those more fortunately situated.

**Seaside Convalescent Hospital** (Seaford, Sussex), 6, Bank-buildings, 1, Long-acre, W.C. Secretary, Mr. Frank Maitland.—There are no more useful institutions than those which provide for hospital patients and other sick persons from the time when disease has ceased and health has to be restored. Amongst the most useful of convalescent hospitals is that at Seaford, where an annual subscription of £1 1s., or a donation of £21, entitles the giver to an admission order for one patient, available for four weeks each year. For some unaccountable reason the managers find it very difficult to secure adequate funds to enable them to keep all the beds occupied, and we heartily commend this institution to the favourable consideration of our readers and philanthropists generally.

**Thames Church Mission,** 31, New Bridge-street, E.C. Secretary, Rev. H. Bloomer.—This mission is under the patronage of the Bishop of London, Sir James Whitehead, Bart., being the president. Of all missionary societies, the Thames Church Mission probably does the largest amount of work of a most useful kind in the quietest and least ostentatious way. We have known those connected with it for many years, some of whom have long since passed to their rest, and we can say that throughout our experience the same zeal and devotion has characterised its conduct. Sailors, bargemen, emigrants, passengers, watermen, and others throughout the Port of London have much cause for thankfulness that this mission exists, and that its missionaries are continuously employed to their great comfort and advantage. Anyone who will pause to think for a moment what the extent of the Port of London in reality is must be empressed by a feeling of admiration for the courage of those who conduct this mission, and by a sincere desire to aid its administration by contributing to its funds.

